# Synchronous gastric adenocarcinoma and gastrointestinal stromal tumor (GIST) of the stomach: A case report

**DOI:** 10.1186/1477-7819-9-60

**Published:** 2011-05-26

**Authors:** Theodosios Theodosopoulos, Dionysios Dellaportas, Vasiliki Psychogiou, Konstantinos Gennatas, Agathi Kondi-Pafiti, Georgios Gkiokas, Ioannis Papaconstantinou, Georgios Polymeneas

**Affiliations:** 1 2nd Department of Surgery, University Hospital Aretaieion, Athens, Greece; 2 1st Department of Pathology, University Hospital Aretaieion, Athens, Greece; 3 Department of Oncology, University Hospital Aretaieion, Athens, Greece

**Keywords:** gastric adenocarcinoma, gastrointestinal stromal tumor (GIST)

## Abstract

Gastrointestinal stromal tumors (GISTs) are rare mesenchymal neoplasms of the gastrointestinal tract (1%), and stomach is the most common location involved. However, the co-existence of gastric adenocarcinoma and GIST is very rare. A case of an 80-year-old male with a simultaneous presentation of a gastric adenocarcinoma and GIST is presented. Various hypotheses have been proposed in order to explain this rare simultaneous development, but even though it's cause has not been proven yet.

## Background

Gastrointestinal stromal tumors (GISTs) the commonest non-epithelial tumors, are rare mesenchymal neoplasms of the gastrointestinal tract, accounting only for 1% of all gastrointestinal malignancies [1-3]. Interstitial cells of Cajal, which are responsible for gut motility, is believed to be GIST's precursors, because both express the receptor tyrosine kinase KIT (c-KIT) [4,5]. We present a rare case of synchronous occurrence of adenocarcinoma of the stomach in an 80-year-old male and GIST.

## Case presentation

**Figure 1:**
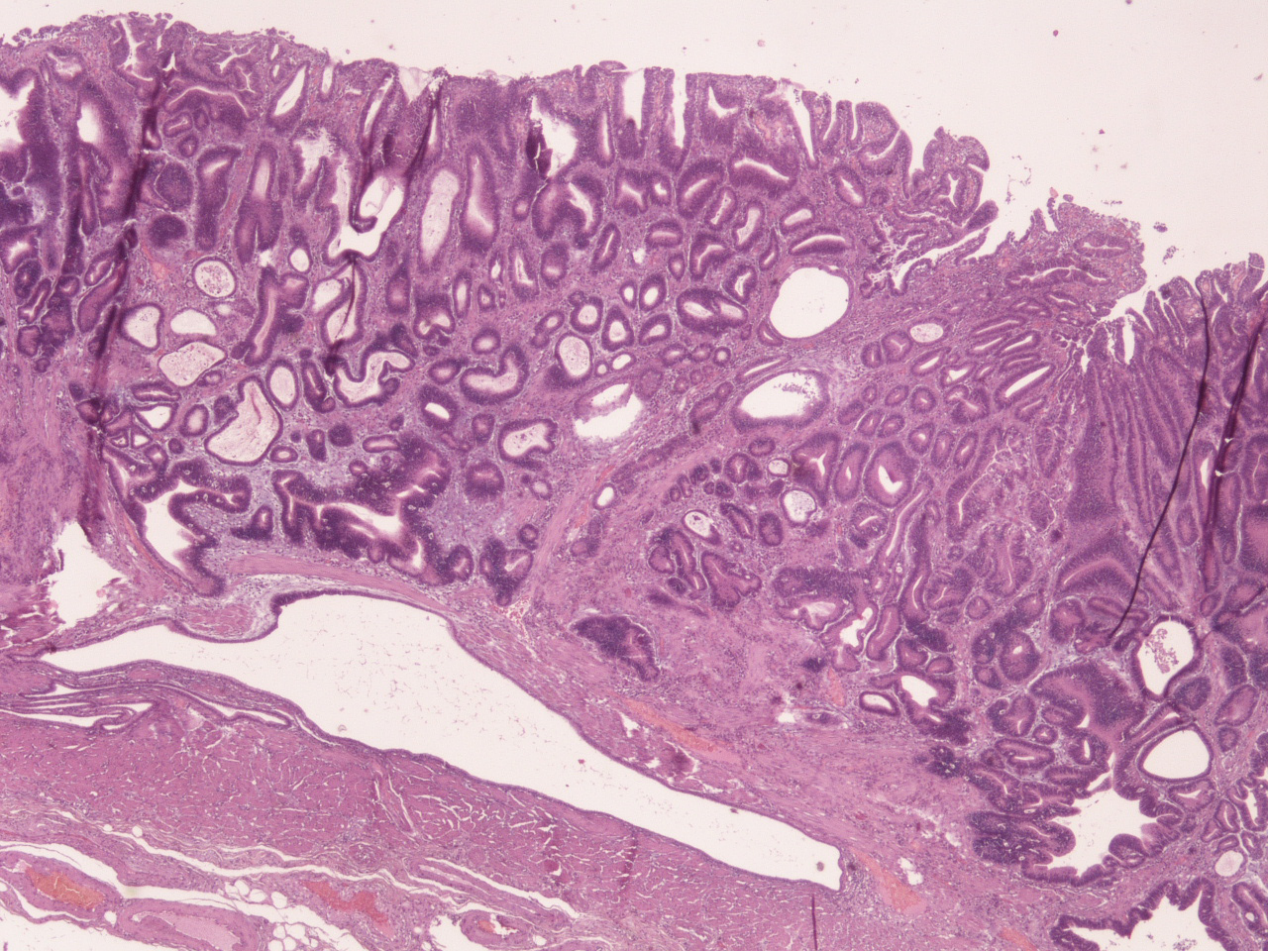
Microscopic image of adenocarcinoma

**Figure 2:**
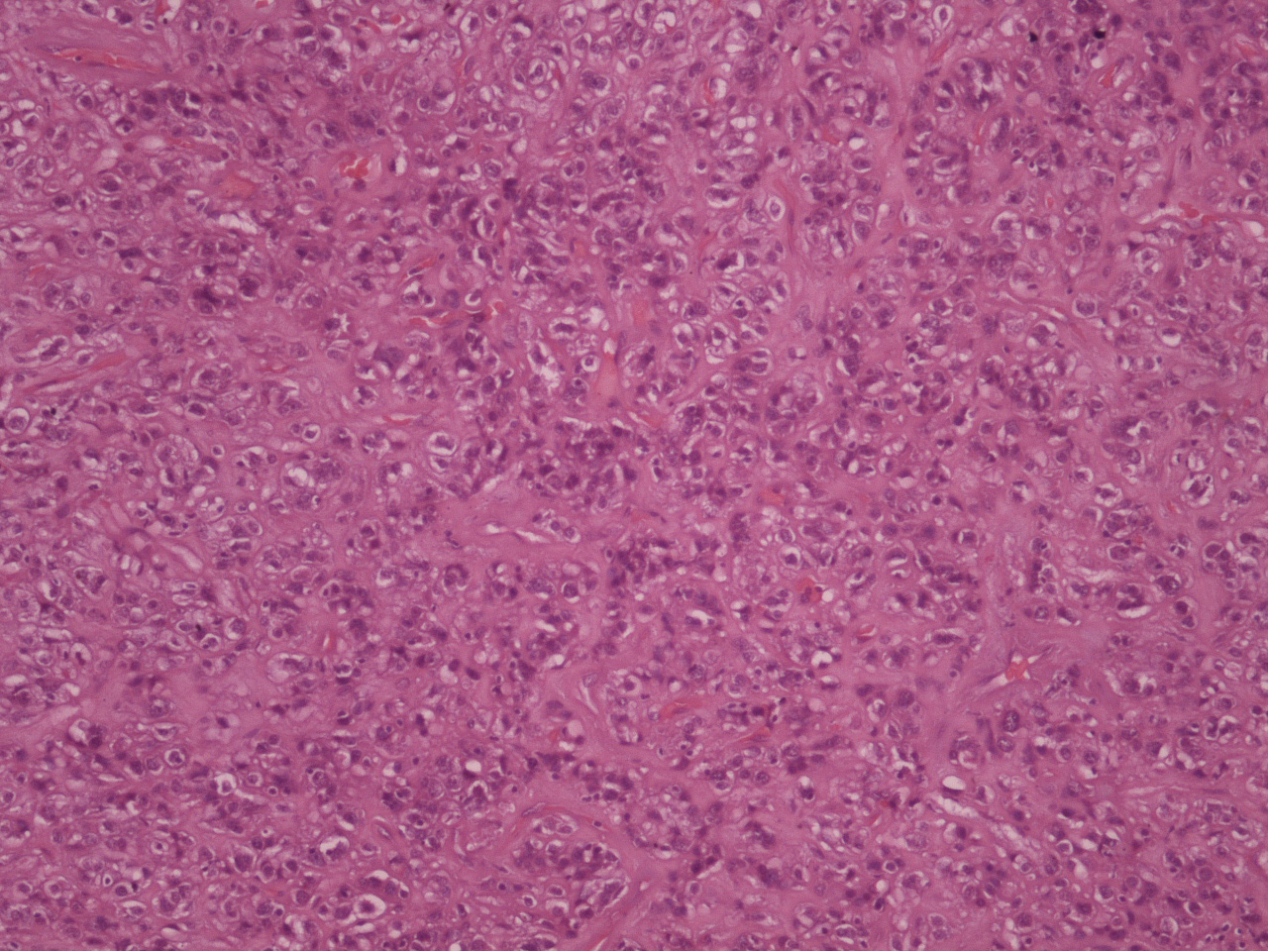
Microscopic image of GIST

An 80-year-old male was admitted to our hospital complaining about epigastric discomfort after meals, nausea and weight loss of about 8 kg during the last three months. Mild anaemia was present, but physical examination and other laboratory tests were unremarkable. Esophagogastroscopy revealed an ulcerative mass in the gastric antrum on the lesser curvature measuring 4 × 6 cm. Pathology report of the endoscopic biopsies revealed a well differentiated intestinal type gastric adenocarcinoma. Chest and abdominal CT-scan for staging demonstrated no sites of distant metastasis. The patient underwent subtotal gastrectomy and Billroth-II gastrojejunal anastomosis. During laparotomy a second nodule was palpated about 3 cm proximal to the neoplasm at the lesser curvature. Pathology examination confirmed the presence of a well differentiated intestinal type gastric adenocarcinoma measuring 6,5 cm in diameter, infiltrating the submucosa of the stomach (Figure 1), while none of the 21 resected lymph nodes contained metastasis. The second lesion, however, was a 3 cm GIST with intermediate malignant potential, having six mitoses per 50 high power fields, but with severe nuclear atypia and c-kit positive (Figure 2). The postoperative course was uneventful and the patient was discharged on the eighth postoperative day. The patient received imatinib as adjuvant therapy for the GIST, according to the international guidelines for GIST's risk stratification [1]. One year later on his follow up visit he remains clinically and radiographically disease free.

GISTs are the most common non-epithelial tumors of the digestive tract accounting for the 1% of all gastrointestinal malignancies and stomach is the most common location involved (40-60%). They were previously reported as leiomyomas, leiomyosarcomas, schwannomas, but the last decade and after the implementation of immunohistochemicals stains and electron microscopy, these tumors have been recognized as distinct pathological entity [1-6]. These tumors are believed to originate from interstitial cells of Cajal or their precursors, because both strongly express the c-KIT protein (CD117), which is a type III tyrosine kinase receptor encoded by the c-kit proto-ongogene [7]. These tumors often express BCL-2 (80%), CD34 (70%), SMA (35%), S-100 (10%) and desmin (5%) [8]. Based on that expression GISTs are the first kind of tumors for which targeted therapy was introduced, using imatinib, which is an inhibitor of receptor tyrosine kinases including KIT, platelet-derived growth factor receptors (PDGFRs), colony stimulating factor 1 receptor (c-FMS), breakpoint cluster region and abl gene fusion protein (BCR-ABL) and specifically blocks the adenosine-5'-triphosphate (ATP) binding site [9]. Rare cases of synchronous presentation of gastric adenocarcinoma and GIST have been previously reported [10-13], but no convincing explanation is still given for this coexistence. In our case gastric adenocarcinoma and GIST's site of occurrence were different, however, collision tumors have also been reported [14]. Simple coincidence could be the most obvious explanation, but gene mutations or influenced neighboring stomach tissues by the same carcinogen are another two hypothesis reported in the literature [10-15]. A combined genetic deregulation seems to be involved in the pathogenesis of these two entities. Surgical excision is the therapeutic approach for both of them following oncologic principles. The postoperative adjuvant therapy should include either chemotherapy for the adenocarcinoma, depending on the pathology report and disease stage and/or imatinib for the GIST depending on the risk category stratification according to the international guidelines.

## Conclusions

Concurrent existence of gastric adenocarcinoma and GIST is a rare case and proven relation of this synchronous development has not been established. High clinical suspicion during laparotomy for another reason is required in order to detect GISTs, because they are asymptomatic and incidental findings most of the times. Surgical excision is the mainstay of therapy and further research is needed for explaining this simultaneous tumor development, if there is such.

## Consent

Written informed consent was obtained from the patient for publication of this case report and any accompanying images. A copy of the written consent is available for review by the Editor-in-Chief of this journal.

## Competing interests

The authors declare that they have no competing interests.

## Authors' contributions

TD designed the structure of the article, DD and VP performed research and wrote the paper, KG and AKP revised the article, GG and IP helped in coordination and to draft the manuscript, and GP gave the final approval of the version to be published. All authors read and approved the final manuscript.
